# Central New York Dental Association

**Published:** 1865

**Authors:** S. B. Palmer

**Affiliations:** President


### CENTRAL NEW YORK DENTAL ASSOCIATION.

At the last meeting of that Society, held in Syracuse, the time for holding its meeting was changed from the Second Tuesday of May and November to the Third Tuesday of June and December.

The Second Annual Meeting will be held in Skaneateles on Tuesday, June 13th, commencing at 10 o'clock, A. M.

S. B. PALMER. President.

